# Emotional Pain and Emotional Support in Nursing Homes

**DOI:** 10.1192/j.eurpsy.2026.11651

**Published:** 2026-07-31

**Authors:** M. E. Van Bogaert, H. Lahaye, B. Yachou, K. Gillis

**Affiliations:** 1 Centre for Health for Older People (HOPE), Odisee University college, Sint Niklaas; 2 Centre for Resarch and Innovation in Care, University of Antwerp, Antwerp, Belgium

## Abstract

**Introduction:**

Psychological pain is probably inherent to the human existence. When we get older, the likelihood of experiencing psychological pain may increase due to the accumulation of experienced loss and failure. When people move to a nursing home, residents may experience difficulties with dealing with their psychological pain. Given the role of psychological pain in residents’ quality of life, early detection and adequate support is important to promote existential health and well-being.

**Objectives:**

To have a better understanding of nursing home residents’ psychological pain and frustrated needs, their longings and their strategies to regulate their emotions and psychological pain and to provide a definition of emotional support to strengthen caregivers’ practice in the recognition and answering of unmet needs when people experience psychological pain.

**Methods:**

Qualitative design was used based on semi-structured interviews. Date were analyzed both inductively and deductively, based on the definition of psychological pain (Meerwijk & Weiss. J. Loss Trauma 2011;16 5) and the Senses Framework (Nolan et al. GRIP 2006; No2). Additionally, a definition for emotional support was formulated using the Delphi method in three steps to establish the comprehensiveness and the extent of agreement of the definition (CVI>80%).

**Results:**

All of the 27 participants have experienced psychological pain in their lives. Their longings and coping strategies differed. A sense of belonging, a sense of significance and a sense of continuity were the three most expressed unmet needs. While almost all participants indicated they longed for contact and visits from their families and friends, some participants also described the relationship they had with staff as valuable. Participants used acceptance of and adaptation to the situation, speaking up on their feelings to experience relief or in contrary not talking about their pain to avoid negative emotions, distraction, reminiscing to the past and asking actively for emotional support as coping strategies. Emotional support was defined as the listening of someone to their story with attention and without judgement and the being present with or without words, as the acknowledgment and the given space to all their emotions, the carry of difficulties and the share of their joy. Where needed, others support in how they cope with their emotions, attuned to who they are.

**Conclusions:**

The experience of psychological pain can be considered as part of nursing home residents’ emotional life. Understanding emotional support and insights in common coping strategies to deal with psychological pain, may support caregivers to promote existential health and well-being of older adults in nursing homes.

**Disclosure of Interest:**

None Declared

